# S09-1 Which transport policies increase physical activity of the whole of society? A Systematic Review

**DOI:** 10.1093/eurpub/ckac093.045

**Published:** 2022-08-29

**Authors:** Joanna Zukowska, Anna Gobis, Piotr Krajewski, Agnieszka Morawiak, Romanika Okraszewska, Catherine B Woods, Kevin Volf, Liam Kelly, Peter Gelius, Sven Messing, Sarah Forberger, Jeroen Lakerveld, Enrique García Bengoechea, on behalf of the PEN consortium

**Affiliations:** 1 Faculty of Civil and Environmental Engineering, Gdansk University of Technology, Poland; 2 Physical Activity for Health Research Cluster, Health Research Institute, Department of Physical Education and Sport Sciences, University of Limerick; 3 Friedrich-Alexander-Universität Erlangen-Nürnberg, Erlangen, Germany; 4 Leibniz Institute for Prevention Research and Epidemiology – BIPS, Bremen, Germany; 5 Amsterdam UMC, VU University Amsterdam, Department of Epidemiology and Data Science, Amsterdam Public Health research institute, De Boelelaan 1089a, Amsterdam, the Netherlands; 6 Upstream Team, www.upstreamteam.nl, Amsterdam UMC, VU University Amsterdam, De Boelelaan 1089a, Amsterdam, the Netherlands

**Keywords:** physical activity, benchmarking, policy, implementation, sport, transport, mass media

## Abstract

**Background:**

There is strong evidence of the links between car-dependence and the global physical inactivity epidemic. If eliminated, physical inactivity would remove between 6% and 10% of major non-communicable diseases that are the leading cause of death globally, killing 38 million people each year. Research consistently shows that unlike passive transport (e.g. driving a car), active transport (i.e., walking, cycling) is associated with higher total daily physical activity (PA). While there are public policies that support PA in transport and, as a result, overall PA levels, the specific quantitative effect of such policies on PA behaviour has not been sufficiently investigated. The aim of this systematic review is to determine the level and type of evidence for policies in the area of transport that contribute to higher PA levels of society at large.

**Methods:**

Six databases (MEDLINE (Ebsco), SportDiscus, Cinahl, Cochrane library, Web of Science, and Scopus) were searched for key concepts of policy, transport, evaluation and PA. Methodological quality was assessed using standardised tools. The strength of the evidence of policy impact was described based on pre-determined categories of positive, negative, inconclusive or untested.

**Results:**

17 of 2,549 studies were included in the data synthesis. The authors identified three main transport policy areas with 60 individual policy actions that had a direct or indirect effect on PA. The policy areas were: convenient transport infrastructure development, active travel promotion and shift of transport mode. These areas correspond to Haddon's methodological approach of the transport system division in to three elements (human, vehicle, road). More than half of the policy actions identified (53%) had a positive effect on PA. Study quality ratings were moderate to good.

**Conclusions:**

PA levels can be increased by implementing policies that provide convenient, safe, and connected walking and cycling infrastructures, promote active travel and give strong support to public transport. There is also clear evidence that active travel policies work best when implemented in a comprehensive way (very often as a combination of several policies). This may include infrastructure and facility improvements as well as educational programmes to achieve substantial shifts towards active modes of travel.

